# The Effect of Retrieval Focus and Emotional Valence on the Inferior Frontal Cortex Activity during Autobiographical Recollection

**DOI:** 10.3389/fnbeh.2013.00192

**Published:** 2013-12-16

**Authors:** Ekaterina Denkova, Sanda Dolcos, Florin Dolcos

**Affiliations:** ^1^Alberta Cognitive Neuroscience Group, University of Alberta, Edmonton, AB, Canada; ^2^Psychology Department, University of Illinois at Urbana-Champaign, Urbana, IL, USA; ^3^Neuroscience Program, University of Illinois at Urbana-Champaign, Urbana, IL, USA; ^4^Beckman Institute for Advanced Science and Technology, University of Illinois at Urbana-Champaign, Urbana, IL, USA

**Keywords:** episodic memory, emotional valence, retrieval goal, inferior frontal gyrus, insula

## Abstract

Although available evidence points to a role of the inferior frontal cortex (IFC) in both emotion processing and autobiographical memory (AM) recollection, it is unclear what the role of this region is in emotional AM recollection. The present study investigated whether IFC activity can be influenced by manipulations of the retrieval focus (emotional vs. non-emotional) and whether this influence is similar for AMs with positive and negative emotional valence. Participants were asked to focus either on emotional (*Emotion* condition) or on non-emotional contextual (*Context* condition) details during the elaboration of positive and negative AMs, while fMRI data were collected. The study yielded two main findings: (1) Focusing on Emotion compared to Context during AM recollection was associated with increased activity in bilateral IFC, for positive AMs, whereas negative AMs produced similarly high IFC activity during Emotion and Context conditions; (2) There was a hemispheric dissociation in the IFC linked to the experiencing of emotion and the focus of AM recollection, such that the left IFC activity correlated positively with the subjective re-experience of emotion during the Emotion condition, whereas the right IFC activity correlated negatively with the subjective re-experience of emotion during the Context condition, for both positive and negative AMs. Overall, the present findings suggest that IFC’s involvement during the recollection of emotional AMs is susceptible to manipulations of the retrieval focus only in the case of positive AMs, and that this region plays a role in both the enhancement and inhibition of emotional experience during AM recollection.

## Introduction

There is considerable evidence that the inferior frontal cortex (IFC) plays an important role in language (Poldrack et al., 1999), cognitive control (Badre, 2008), memory (Thompson-Schill et al., 1997; Fletcher and Henson, 2001), emotion processing (Wager et al., 2008; Lindquist et al., 2012), and emotion regulation (Ochsner et al., 2012), possibly through its involvement in operations such as language unification (Hagoort, 2005), controlled retrieval (Badre and Wagner, 2007), selection among competing alternatives (Thompson-Schill et al., 1999; Moss et al., 2005; Grindrod et al., 2008), integration of information (Fuster, 2002), and response inhibition (Aron et al., 2004). Of particular interest for the present investigation, evidence derived from separate lines of research points to a pivotal role of the IFC in memory retrieval (Greenberg et al., 2005; Badre and Wagner, 2007) and in emotion processing (Wager et al., 2008; Lindquist et al., 2012). The goal of the present investigation is to elucidate the role of IFC in the retrieval of emotional autobiographical memories (AMs) according to the retrieval focus and the valence of memories.

Functional neuroimaging evidence from separate lines of investigations suggests a role of the IFC in both the enhancement and inhibition of emotion processing (Hooker and Knight, 2006; Dolcos et al., 2011; Ochsner et al., 2012; Iordan et al., 2013). Studies investigating the influence of emotion on memory have provided evidence for a role of the IFC in enhancing the effect of emotion on memory formation (Dolcos et al., 2004) and in diminishing the impact of negative goal-irrelevant emotional distraction on working memory (Dolcos et al., 2006, 2008, 2013). Studies investigating the neural correlates of emotion control have pointed to the IFC’s contribution to voluntary up- and down-regulation of emotion (Ochsner et al., 2004; Kim and Hamann, 2007). The evidence from these investigations is based nearly exclusively on externally triggered emotions in experimental settings (e.g., by viewing emotional pictures), most of the time negative, rather than on internally triggered emotions (e.g., recall of emotional personal memories). Here, we investigate the role of IFC in the processing of internally triggered emotions associated with the recollection of emotional AMs.

Internally triggered emotions are more ecologically valid and can generate stronger emotional responses than those produced by external stimulation in experimental settings (Salas et al., 2012). Also, the former are at the basis of maintaining affective disorders, such as depression and post-traumatic stress disorder (Brewin et al., 1999; Rubin et al., 2008), which are characterized by an increased focus toward negative personal memories and/or inhibition of positive ones (Werner-Seidler and Moulds, 2011). Recollection of emotional AMs has been linked, among other brain regions, to the involvement of IFC (Markowitsch et al., 2000). The more ventral portion of the IFC, part of the temporo-frontal junction interconnected through the ventral branch of the uncinate fascicle, has been attributed a crucial role in “synchronizing emotional and factual components of the personal past” during remembering (Brand and Markowitsch, 2008; p. 326; Markowitsch, 1995; Brand and Markowitsch, 2006). IFC’s involvement has also been found in “non-emotional” AM studies (Conway et al., 1999; Piolino et al., 2004; Greenberg et al., 2005; Daselaar et al., 2008). Typically, this region has been associated with successful memory retrieval, which involves strategic search and selection of appropriate information and monitoring of the veracity and cohesiveness of the recollected memory (Svoboda et al., 2006; Badre and Wagner, 2007). It is not clear, however, whether IFC’s involvement during the recollection of emotional AMs can be influenced by the focus of retrieval (emotional vs. non-emotional), and whether this influence is similar for positive and negative AMs. Given that positive and negative AMs may be governed by different mechanisms and lead to different outcomes (Denkova et al., 2012), and that IFC appears to be more involved for negative AMs (Markowitsch et al., 2003), it is important to clarify the role of emotional valence in the retrieval of emotional AMs. While negative memories have received, overall, much more attention in the literature, there is also evidence highlighting the importance of positive memories in promoting personal self-esteem and overall positive mindset (Diener and Seligman, 2002; Fredrickson, 2004; D’Argembeau and Van der Linden, 2008; Denkova et al., 2012), and their beneficial effects in depressed people (Dalgleish et al., 2013).

The main goal of the present study was to investigate the involvement of the IFC during the recollection of emotional AMs, linked to the focus of retrieval (emotional vs. non-emotional) and the valence of memories (positive vs. negative). For this purpose, fMRI data were recorded while participants were cued to focus either on emotional (*Emotion* condition) or on non-emotional contextual (*Context* condition) details, during elaboration of highly emotional positive and negative AMs. Based on the extant evidence, we made the following predictions. General sensitivity of the IFC to manipulations of the retrieval focus should be reflected in differential engagement of this region in the *Emotion* and *Context* conditions, for both positive and negative AMs, such that increased IFC activity during the *Emotion* condition would be associated with enhanced emotional experience. However, it is also expected that retrieval focus may also result in specific sensitivity of the IFC response linked to the valence of AMs, possibly reflecting the enhancement of positive emotions and the inhibition of negative emotions.

## Materials and Methods

### Participants

Analyses were performed on data from 17 right-handed native English speaking young adults (6 men; age range 18–46, mean = 26.06 years, SD = 7.20), who provided written informed consent and received payment for their participation. The experimental protocol was approved by the Institutional Health Research Ethics Board.

### Collection and selection of emotional autobiographical memories

Personal memories were elicited from each participant during an interview performed approximately 5 weeks prior to the fMRI session using an autobiographical memory questionnaire (AMQ) (Denkova et al., 2012). The AMQ comprised a list of 115 verbal cues for distinct life events (e.g., “the birth of a family member,” “being hospitalized”); for each of them, participants were asked to remember a unique episode from their life and to provide a brief description of the memory, which was then used as a personalized memory cue during the fMRI scanning. Phenomenological characteristics of each event were assessed by asking the participants to date the memory and rate it on several Likert scales including Emotional Valence (using a 7-point scale: −3 = very negative, 0 = neutral, and +3 = very positive), Emotional Intensity, Personal Significance, Vividness, the amount of Contextual Details, and the Frequency of Retrieval (all of the latter used a 7-point scale: 1 = not at all, 7 = extremely). For each participant, we selected the 40 most emotional memories (20 positive and 20 negative), based on the emotional ratings. Half of the selected memories, with an equal proportion of positive and negative, were assigned to the *Emotion* condition, and the other half of AMs were assigned to the *Context* condition.

### fMRI tasks

#### The autobiographical memory tasks

(i) In the *Emotion* focus condition, participants were instructed to remember the specific event and focus on the emotional aspects of their memories, including associated sensations and feelings (e.g., butterflies in the stomach, palpitations). (ii) In the *Context* focus condition, participants were instructed to remember the specific event and focus on the contextual aspects of their memories, by retrieving as many contextual details as possible (e.g., about where and when the event occurred). Each memory cue was preceded either by the instruction cue “Remember Emotion” (for the Emotion condition), or “Remember Context” (for the Context condition). After recollection, each event was rated on three five-point Likert scales including Emotional Intensity, Vividness, and Reliving (1 = very low; 5 = very high).

#### The semantic memory control task

In line with other AM studies (Greenberg et al., 2005; Young et al., 2013), we also used a control condition involving semantic memory (SM) retrieval, such as the generation of exemplars from different semantic categories (e.g., sports, vegetables) (Battig and Montague, 1969). Each semantic category cue was preceded by the instruction cue “Generate Examples.” To be consistent with AM conditions, each exemplar generation was rated on three five-point Likert scales including Vividness, Difficulty of the task, and approximate Number of the recalled items.

### fMRI design

The AM and SM conditions had the same general structure (Denkova et al., 2011). Each trial began with an instruction screen for 2 s, immediately followed by a memory cue for 4 s. After the cue offset, a fixation screen was presented for 10 s during which participants elaborated their personal memories or generated exemplars. The end of the retrieval period was marked by an instruction screen for upcoming ratings, for 1.5 s. Then, each of the three ratings was presented for 2.5 s and in a counterbalanced order across trials. The ratings were followed by an inter-trial interval of variable duration (2–9 s, average = 6 s), before the beginning of the next trial.

### MRI data collection

MRI data were recorded using a 1.5-T Siemens Sonata scanner. The anatomical images were 3D MPRAGE anatomical series [repetition time (TR) = 1600 ms, echo time (TE) = 3.82 ms, field of view (FOV) = 256 mm × 256 mm, number of slices = 112, voxel size = 1 mm × 1 mm × 1 mm]. The functional images consisted of series of images acquired axially using an echoplanar sequence (TR = 2000 ms, TE = 40 ms, FOV = 256 mm × 256 mm, number of slices = 28, voxel size = 4 mm × 4 mm × 4 mm).

### fMRI data analysis

Statistical analyses, performed with SPM2 (Statistical Parametric Mapping), were preceded by the following pre-processing steps: Quality Assurance, TR Alignment, Motion Correction, Coregistration, Normalization, and Smoothing (8 mm full-width half maximum isotropic Kernel). At the individual level, each event was modeled by the canonical hemodynamic response function (*hrf*) and its temporal derivate. The *hrf* was time-locked to 2 s (1 TR) following the onset of the memory cues, in the Emotion and Context AM conditions, and 1 s (0.5 TR) after the onset of the category cue, in the SM condition to allow time for reading the cues. This procedure was guided by the present RT data, which showed that the recognition of the AM cue and beginning of retrieval occurred at an average RT of 1.67 s (±0.44), and the beginning of the exemplar generation in the SM condition occurred at an average RT of 1.03 s (±0.40). This procedure allows comparisons of the fMRI signal associated with AM and SM retrieval, by accounting for differences in the timing of retrieval operations and memory identification, and is consistent with the procedure used in previous neuroimaging studies of AM retrieval (Addis et al., 2007). Individual contrasts were computed directly between the different AM event types (e.g., Emotion Positive vs. Context Positive, Emotion Negative vs. Context Negative). These individual contrasts were then entered into group-level *t* tests, to perform random-effects analyses.

The effects of retrieval focus were investigated by comparing AMs with Emotion focus and AMs with Context focus separately for positive and negative AMs. The interaction effects of retrieval focus and valence were investigated using paired *t* tests [e.g., (Emotion Positive vs. Context Positive) vs. (Emotion Negative vs. Context Negative)], whose outputs were inclusively masked with the direct contrast of interest (e.g., Emotion Positive vs. Context Positive), to ensure that the interaction difference is due to an existing increased difference in the contrasts of interest. Finally, to investigate whether activity in IFC according to the retrieval focus and valence is linked to the self-reported re-experience of emotion, linear regression analyses were performed between overall IFC activity in each AM condition (i.e., Emotion Positive vs. baseline; Emotion Negative vs. baseline; Context Positive vs. baseline and Context Negative vs. baseline) and the corresponding self-reported emotional ratings.

Activity in regions of interest was investigated using adapted anatomical masks from the Wake Forest University Pick Atlas toolbox. The threshold was set up at *p* < 0.001 for the direct contrasts and at *p* < 0.05 for the interactions and correlations; the extent threshold was of five contiguous voxels in all analyses. The interaction maps were masked inclusively with the corresponding direct contrast set up at *p* < 0.001. Activations in other brain regions, including basic emotion and memory-related medial temporal lobe (MTL) brain areas are reported in a previous report (Denkova et al., 2013). In short, these findings revealed increased activity for positive AMs in the amygdala (AMY) and hippocampus, and in other brain regions, including lateral temporal and prefrontal cortices. Given the similarity of patterns observed in the AMY and IFC, we further investigated the relationship between activity in these two regions, by performing linear regression analyses between IFC activity for Emotion vs. Context contrast (extracted from peak voxels showing significant differences in activation, in the right and left IFC, BA 47) and brain activity in the AMY.

## Results

### Behavioral results

#### Increased re-experiencing of emotion during emotion focused retrieval for both positive and negative AMs

Repeated-measures ANOVA revealed a focus × ratings interaction [*F*_(1, 16)_ = 4.12, *p* = 0.03], driven by an increase only for the emotional intensity ratings of AMs retrieved with an emotional focus and the absence of significant differences in the other ratings (Reliving and Vividness ratings). The increase was significant for both positive (3.21 vs. 3.03, *p* = 0.02) and negative (3.38 vs. 3.07, *p* = 0.003) AMs.

### fMRI results

#### Increased IFC activity for emotion compared to context focus for positive AMs

**Figure 1:**
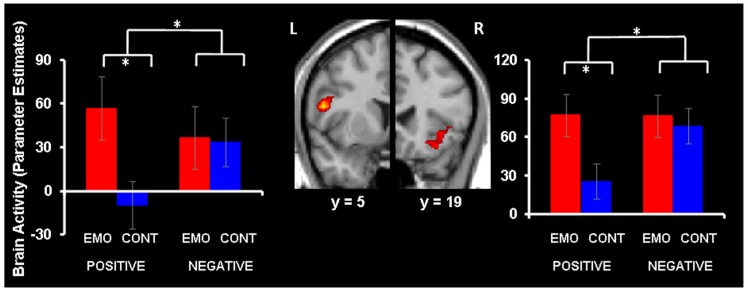
**Increased IFC activity for emotion compared to context focus for positive AMs**. Focusing on Emotion (EMO) compared to focusing on Context (CONT) led to increased activity in bilateral IFC (red blobs) for positive AMs, whereas negative AMs produced similar IFC activity during EMO and CONT conditions. The interaction map is superimposed on a high resolution brain image displayed in a coronal view (with *y* indicating the Talairach coordinates on the anterior-posterior axis of the brain). For illustration purpose, the interaction map set up at *p* < 0.05 is inclusively masked with the direct contrast set up at *p* < 0.05; the effects are also observed when the mask is set up at *p* < 0.001 (Table 1). The bar graphs represent the contrast estimates extracted from representative voxels in the left and right IFC, respectively. The error bars correspond to the standard errors of the means. L, Left; R, Right.

Focusing on Emotion compared to Context led to increased activity in bilateral IFC (BAs 44 and 47) for positive memories, but similarly high IFC engagement under the Emotion and Context foci was observed for negative AMs (see Figure 1 and Table 1). These effects were confirmed by a repeated-measures ANOVA performed on the extracted signal, which, in the left IFC (BA 44), revealed a significant valence × focus interaction [*F*_(1, 16)_ = 15.93, *p* = 0.001]. This interaction was driven by a significant increase in the Emotion compared to the Context condition, for positive (*p* < 0.001) but not for negative (*p* = 0.80) AMs. Similarly, the effect in the right IFC (BA 47) was confirmed by a repeated-measures ANOVA revealing a significant valence × focus interaction [*F*_(1, 16)_ = 7.26, *p* = 0.015], which was driven by a significant increase in the Emotion compared to the Context condition for positive (*p* < 0.001) but not for negative (*p* = 0.50) AMs.

**Table 1:** **Significant activations and correlations linked to the retrieval focus and the emotional valence of memories**.

IFC (BA)	Side	Talairach coordinates			*t* Score	Cluster size
		*x*	*y*	*x*		
**DIRECT CONTRASTS**						
Emotion Positive vs. Context Positive						
IFG (44)	L	−55	12	10	4, 91	8
IFG (47)	R	40	27	−1	4, 90	22
IFG (47)	L	−36	19	−8	4, 85	28
**INTERACTIONS**						
(Emotion Positive vs. Context Positive) vs. (Emotion Negative vs. Context Negative)						
IFG (44)	L	−55	12	14	3, 99	7
IFG (47)	R	32	23	−15	2, 69	12
**CORRELATIONS**						
**A. Positive correlations**						
Emotion (Emotion Positive ∩ Emotion Negative)						
IFG (47)	L	−32	15	−11	3, 13/2, 45	7
IFG (46)	L	−48	28	17	2, 14/2, 07	6
Context (Context Positive ∩ Context Negative)						
IFG (46)	L	−51	32	9	2, 61/2, 04	12
**B. Negative correlations**						
Context (Context Positive ∩ Context Negative)						
IFG (47)	R	24	35	−8	3, 42/2, 01	5

*Significant activations and correlations resulting from ROI analyses are reported. For direct contrasts, a threshold of *p* < 0.001 was used. For interactions, a threshold of *p* < 0.05 was used and further inclusively masked with the corresponding direct contrast set up at *p* < 0.001. For correlations, a threshold of *p* < 0.05 was used, and the overlaps between positive and negative AMs within each focus are presented. A cluster size of five contiguous voxels was used for all analyses. IFG, inferior frontal gyrus; BA, Brodmann’s area; L, Left, R = Right*.

#### Hemispheric dissociation in the IFC linked to the experiencing of emotion and the focus of AM recollection

**Figure 2:**
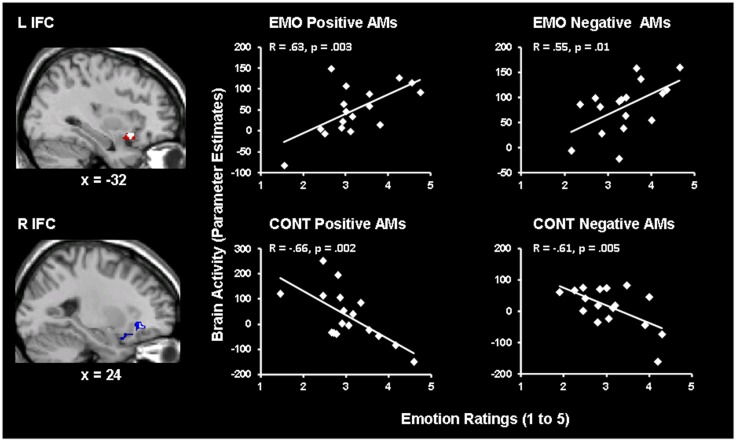
**Hemispheric dissociation in the IFC linked to the experiencing of emotion and the focus of AM recollection**. Activity in the left IFC correlated positively with emotional ratings in the Emotion (EMO) condition, for both positive and negative AMs, whereas activity in the right IFC correlated negatively with emotional ratings in the Context (CONT) focus, for both positive and negative AMs. The correlation maps in the left and right IFC are superimposed on high resolution brain images displayed in sagittal views (with *x* indicating the Talairach coordinates for the left/right hemispheres of the brain). The white blobs represent the areas where there are overlapping voxels for both positive and negative AMs, which are superimposed on larger areas showing positive (red) or negative (blue) co-variations either for positive or for negative AMs. The scatterplots are based on contrast estimates of the IFC activity for each condition, as extracted from the peak voxels of the areas showing the co-variation with the corresponding emotional ratings. L, Left; R, Right.

Brain-behavior correlation analyses revealed opposing patterns of co-variation between activity in the left (showing positive co-variation) and right (showing negative co-variation) IFC and emotional ratings, for Emotion and Context focus, respectively (Figure 2 and Table 1). These effects were common for both positive and negative AMs, as revealed by the conjunction analyses of *Emotion* Positive ∩ *Emotion* Negative and of *Context* Positive ∩ *Context* Negative conditions, respectively. Specifically, activity in a left IFC (BA 47) area, extending to the insula, positively correlated with self-reported re-experience of emotion, for both positive and negative AMs, for the Emotion but not for the Context condition. Similar effects were observed in a more dorsal left IFC (BA 46) area, but they were not specific to the Emotion condition (Table 1). On the other hand, activity in a right IFC (BA 47) area negatively correlated with self-reported re-experience of emotion, for both positive and negative AMs, for the Context but not for the Emotion condition. Overall, these findings suggest a hemispheric dissociation in the IFC linked to the experiencing of emotion and the focus of AM recollection, with the left IFC (BA 47) activity showing specific positive correlations with the subjective re-experience of emotion during the Emotion condition, and the right IFC (BA 47) activity showing specific negative correlations with the subjective re-experience of emotion during the Context condition.

**Figure 3:**
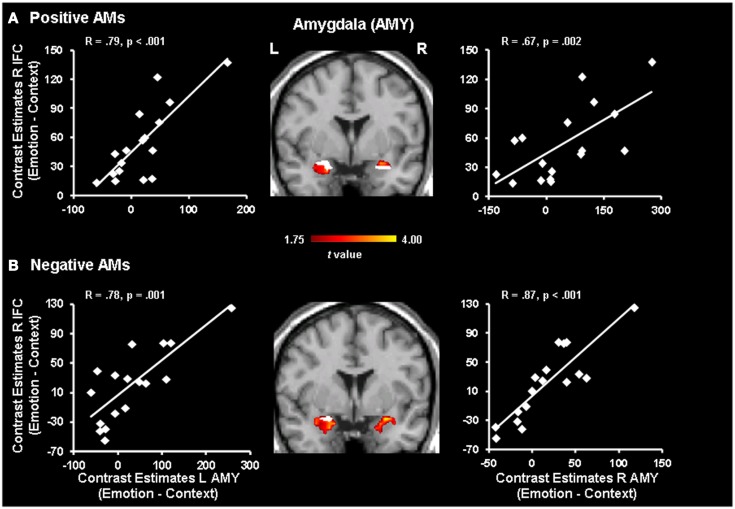
**Positive correlations between activity in the IFC and the AMY**. Activity in the right Inferior Frontal Cortex (IFC) positively correlated with activity in the left and right AMY (red blobs) for positive **(A)** and negative **(B)** AMs, despite the absence of significant differences in the IFC activity between Emotion and Context for the latter. Portions of the AMY showing positive co-variations with the IFC also overlapped (white blobs) with AMY areas showing significant increase in activity for Emotion vs. Context focus. The gradient color bar starts at *p* < 0.05 (*t* = 1.75). Similar patterns of correlations were observed in the left IFC (not shown, see text). The scatterplots are based on contrast estimates for Emotion vs. Context extracted from the peak voxel of the areas showing the co-variation with AMY activity. L, left; R, right.

Further analyses revealed positive co-variations between activity in the IFC and the AMY (Figure 3). For positive AMs, positive co-variations were observed between activity in the right IFC and the right (*x* = 24, *y* = 3, *z* = −14; *R* = 0.67, *p* = 0.002) and left (*x* = −32, *y* = −8, *z* = −13; *R* = 0.79, *p* < 0.001) AMY, as well as between activity in the left IFC and the right (*x* = 32, *y* = −1, *z* = −17; *R* = 0.56, *p* = 0.01) and left (*x* = −28, *y* = −8, *z* = −13; *R* = 0.66, *p* = 0.002) AMY. Of note, portions of the AMY showing positive co-variation with the IFC overlapped with portions of the AMY areas showing significant increase in activity for positive memories with Emotion vs. Context focus reported in Denkova et al. (2013) (Figure 3). Interestingly, similar patterns of positive co-variations between activity in the IFC and AMY were also observed for negative memories, despite the absence of significant differences in the IFC activity between Emotion and Context. Namely, activity in the right IFC positively correlated with activity in the right (*x* = 20, *y* = −1, *z* = −10; *R* = 0.87, *p* < 0.001) and left (*x* = −32, *y* = −4, *z* = −10; *R* = 0.78, *p* = 0.001) AMY, and activity in the left IFC positively correlated with activity in the right (*x* = 20, *y* = −1, *z* = −10; *R* = 0.82, *p* < 0.001) and left (*x* = −32, *y* = −8, *z* = −13; *R* = 0.69, *p* = 0.001) AMY. An overlap between the correlation and activation patterns was observed only in the left AMY (see Figure 3), given that significant differences in activity between Emotion and Context for negative AMs was revealed only in the left AMY (Denkova et al., 2013).

## Discussion

The present study investigated the IFC’s involvement during AM recollection, as a function of the retrieval focus and emotional valence. There were two main findings, which are discussed in turn below.

### Increased IFC activity for emotion compared to context focus for positive AMs

This finding is overall consistent with previous investigations linking IFC’s involvement to enhanced encoding of emotional items (Dolcos et al., 2004), retrieval of emotional AMs (Markowitsch et al., 2000), as well as voluntary up-regulation of positive emotions (Kim and Hamann, 2007). Importantly, the present finding extends the available evidence by showing that activity in the IFC is susceptible to manipulations of the retrieval focus only during the recollection of positive AMs, showing increased activity during retrieval of positive AMs with an emotion focus and decreased activity when the focus is on other non-emotional contextual details. Keeping in mind that IFC is a heterogeneous structure with distinct subregions (Petrides and Pandya, 2002), the increased activity in different IFC subregions in the present study could be interpreted as follows. Given its role in the subjective experience of emotion (Wager et al., 2008), increased activity in BA 47 could reflect the integration and enhancement of emotional experience during autobiographical retrieval. This interpretation is further supported by the positive relationship between activity in the IFC and the AMY, which suggests that IFC’s involvement reflects the integration of emotional information triggered by the AMY and further enhancement of the emotional experience during remembering of AMs with Emotion focus. Given its general role in language production and in inner speech (McGuire et al., 1996; Baciu et al., 1999), particularly during self-referential activities (Morin and Michaud, 2007), of which autobiographical remembering is an essential part, increased activity in the left BA 44 could be linked to a more general reliance on covert speech mechanisms during recollection of positive AMs with Emotion focus.

The present findings suggest that, compared to the emotion focused positive recollections, context focused positive recollections appear to easily lose their emotionality in the absence of an explicit focus on the (re)experienced emotions. This provides a possible explanation for evidence showing that recalling positive memories cannot always reverse negative mood in depressed people (Joormann et al., 2007), and is consistent with recent evidence from clinical studies suggesting that positive memories could potentially alleviate negative mood depending on the way they are processed (Werner-Seidler and Moulds, 2012; Dalgleish et al., 2013). This finding is also consistent with evidence from healthy participants showing that, unlike negative AMs whose retrieval has a strong direct effect on the post-retrieval negative emotional state, retrieval of positive AMs has a weaker and indirect effect on the positive state (Denkova et al., 2012).

In contrast with positive AMs, remembering negative AMs showed similarly high IFC involvement, regardless of the focus of retrieval. This could reflect the engagement of selection and inhibitory operations, necessary to evaluate the relevance of negative emotional information according to the current retrieval goals, and to incorporate and enhance it when relevant (i.e., Emotion focus) and diminish it when not relevant (i.e., Context focus) (Depue et al., 2007). Given the similarity with the response observed in AMY and IFC and their positive co-variation, it is possible that the involvement of some IFC areas reflects the enhancement of the emotional (re)experiencing during the Emotion focus, for both positive and negative AMs, and reduced engagement for positive AMs along with active inhibition of the emotional information (possibly automatically initiated by the AMY) for negative AMs during the Context focus. Overall, these findings suggest that positive AMs can trigger a strong emotional response only if there is an explicit emphasis on the re-experiencing of emotion during remembering, and that this occurs in relationship with activity in the AMY.

### Hemispheric dissociation in the IFC linked to the experiencing of emotion and the focus of AM recollection

These findings suggest a role of the IFC in the modulation (both enhancement and reduction) of emotional experience. The lateralization of these effects could be interpreted in line with previous evidence linking the left frontal cortex to the retrieval of emotional knowledge during up-regulation of emotional response and the right frontal cortex to inhibitory processes during down-regulation of emotional response (Ochsner et al., 2004; Kim and Hamann, 2007). It should be noted that the positive relationship in the left IFC extends to the anterior ventral insula, which is typically co-activated with the IFC (Uddin et al., 2013) and is linked to emotion processing (Chang et al., 2012; Kelly et al., 2012; Lindquist et al., 2012), particularly to the awareness of emotional experiences (Craig, 2009; Zaki et al., 2012). Given that in the Emotion AM condition participants were explicitly instructed to focus on emotional details, including the associated sensations and feelings (e.g., butterflies in the stomach, palpitations), the present findings are consistent with a possible contribution of the Anterior Insula together with the IFC to the enhanced emotional experience during Emotion focus, as reflected in the post-retrieval emotional ratings.

Considering altogether the present findings, it could be speculated that the IFC’s role in enhancing and inhibiting emotion processing may be linked to its more general involvement according to the relevance of processed information to the current goal (Beer et al., 2006). Specifically, when the emotional information is relevant to the current goal (i.e., retrieval focus on emotional details), it can benefit from enhanced processing through the involvement of IFC, whereas when emotional information is not relevant to the current goal (i.e., retrieval focus on non-emotional contextual details), it can be attenuated or inhibited (Conway and Pleydell-Pearce, 2000; Levine and Edelstein, 2009). Finally, the present brain imaging findings, along with the behavioral findings showing an overall reduction of experienced emotion during the Context focus, could also be linked to the manipulation of attentional deployment, as an emotion regulation strategy, which involves a shift in attention away from the emotional aspects of emotion eliciting events by engaging in a competing task (Gross, 2008), or by changing the focus of the recollected memories, as it is the case in the present study.

In summary, the present findings suggest that the IFC’s involvement during the recollection of emotional AMs is susceptible to manipulations of the retrieval focus only in the case of positive AMs, and that this region plays a role in both the enhancement and the inhibition of emotional experience during AM recollection. These findings have direct relevance for therapeutic interventions in affective disorders by pointing to the fact that the increased effectiveness of positive AMs in triggering strong emotional responses, and therefore in alleviating negative mood, occurs only when specific re-experiencing of positive emotions is explicitly emphasized during autobiographical recollection.

## Conflict of Interest Statement

The authors declare that the research was conducted in the absence of any commercial or financial relationships that could be construed as a potential conflict of interest.
